# Daylight Photodynamic Therapy Alone or Combined With Transepidermal Drug Delivery Techniques for Skin Field Cancerization: An Immunohistochemical Study of Epidermal Carcinogenesis and Dermal Remodeling Markers

**DOI:** 10.1111/phpp.70124

**Published:** 2026-10-08

**Authors:** Camila de Oliveira Bento, Luciana Pantaleão, Porphirio José Soares Filho, Ronir Raggio Luiz, Ronaldo Altenburg Odebrecht Curi Gismondi, Maria Claudia Almeida Issa

**Affiliations:** ^1^ Department of Clinical Medicine—Dermatology Fluminense Federal University Niterói Brazil; ^2^ Department of Pathology Fluminense Federal University Niterói Brazil; ^3^ Department of Statistics Federal University of Rio de Janeiro Rio de Janeiro Brazil; ^4^ Department of Clinical Medicine Fluminense Federal University Niterói Brazil

**Keywords:** daylight photodynamic therapy, dermal remodeling, Ki67, p53, skin field cancerization, transepidermal drug delivery

## Abstract

**Background:**

Daylight photodynamic therapy (DL‐PDT) has been combined with transepidermal drug delivery (TED) techniques to enhance photosensitizer penetration. Although studies have suggested improved clinical and histological outcomes with DL‐PDT combined with ablative laser, evidence regarding carcinogenesis and dermal remodeling markers remains limited.

**Objective:**

To evaluate changes in epidermal carcinogenesis and dermal remodeling markers after standard DL‐PDT compared with DL‐PDT combined with TED techniques for facial field cancerization.

**Methods:**

Forty patients were randomized into four groups (*n* = 10): standard DL‐PDT, DL‐PDT with microneedling, DL‐PDT with CO_2_ laser, and DL‐PDT with microdermabrasion. All underwent two treatment sessions 1 month apart using methyl aminolevulinate cream followed by 2 h of daylight exposure. Skin biopsies were obtained at baseline and 3 months after the second session. Immunohistochemistry evaluated p53, Ki67, Collagens I and III, metalloproteinases (MMP‐1, MMP‐3, MMP‐9), and TIMP‐1.

**Results:**

A reduction in the vertical extent of Ki67 expression (from two‐thirds to one‐third of the epidermis) was observed mainly in the microneedling and CO_2_ laser groups, although it was not statistically significant. No significant differences in Ki67 or p53 expression were observed within or between groups. Increased Type III collagen, MMP‐3, and MMP‐9 expression and decreased MMP‐1 and TIMP‐1 expression were observed, with only TIMP‐1 reduction being statistically significant (*p* = 0.041) in the overall analysis.

**Conclusions:**

A trend toward reduced vertical Ki67 expression suggests potential benefit of DL‐PDT combined with microneedling and CO_2_ laser, and favorable changes in dermal remodeling markers were observed overall. These exploratory findings require confirmation in larger randomized studies.

**Trial Registration:** ClinicalTrials.gov: NCT03963765

## 1 Introduction

The concept of skin field cancerization emphasizes the importance of treating not only clinically visible lesions but also the entire photodamaged area, due to the presence of histologically abnormal tissue surrounding actinic keratoses (AKs), which are common premalignant lesions that may progress to squamous cell carcinoma (SCC) [1, 2]. Among the available treatments for field cancerization, photodynamic therapy (PDT) is an effective option, with high efficacy and demonstrated rejuvenating effects at clinical, histological, and molecular levels [3, 4].

Daylight photodynamic therapy (DL‐PDT) is a more recent approach that has demonstrated efficacy comparable to conventional PDT for the treatment of AK and field cancerization, while being associated with minimal discomfort for patients [5]. Our group recently demonstrated improved clinical and histological outcomes with DL‐PDT combined with transepidermal drug delivery (TED) techniques, particularly when associated with CO_2_ laser [6]. However, molecular analyses of these treatment approaches remain limited.

In the present study, we evaluated epidermal markers associated with carcinogenesis, including Ki67 and p53, as well as dermal remodeling markers, including Type I and III collagens, matrix metalloproteinases (MMP‐1, MMP‐3, and MMP‐9), and tissue inhibitor of metalloproteinase‐1 (TIMP‐1).

Our objectives were to evaluate the effects (primary outcome) of DL‐PDT alone and in combination with TED techniques (microneedling, CO_2_ laser, or microdermabrasion), and to compare outcomes among treatment groups (secondary outcome).

## 2 Materials and Methods

This study represents a prespecified exploratory immunohistochemical analysis of a previously published randomized clinical trial evaluating the clinical and histological outcomes of DL‐PDT alone or combined with TED techniques [6].

Forty patients aged 41–74 years, with Fitzpatrick Skin Phototypes II–IV and Glogau Photoaging Grades II–IV, were recruited between February 2017 and February 2018 at a tertiary referral hospital (Antônio Pedro University Hospital, Fluminense Federal University), Niterói, Rio de Janeiro, Brazil. As this was an exploratory study, participants were selected using convenience sampling.

The study was approved by the Research Ethics Committee of Antônio Pedro University Hospital and was registered on ClinicalTrials.gov (NCT03963765). Written informed consent was obtained from all participants before enrollment. Inclusion criteria were age between 40 and 75 years, both sexes, Fitzpatrick Skin Phototypes I–IV, photodamaged facial skin, and the presence of at least one AK lesion on the face. Exclusion criteria were pregnancy, lactation, smoking, history of photosensitivity, malignant neoplasms, collagen diseases, infections, immunosuppression, and any topical treatment or procedure within 3 months before study initiation.

All eligible participants were recruited and enrolled by the study investigators, assigned a study identification number, and registered before randomization. The allocation sequence was subsequently generated using the Random application (Mireia Lluch Ortola, version 1), which assigned participants to one of four treatment groups (10 patients per group):

I—Standard DL‐PDT (control group).

II—DL‐PDT with microneedles.

III—DL‐PDT with CO_2_ laser.

IV—DL‐PDT with microdermabrasion.

All patients underwent two DL‐PDT sessions one month apart. This interval was selected based on previously published clinical protocols and consensus recommendations for the treatment of actinic keratosis and field cancerization using PDT [7]. Patients were instructed to avoid sun exposure for 48 h after each procedure and to apply sunscreen and moisturizer between sessions.

### 2.1 Treatment Protocols

After superficial curettage of the entire face using a dermatological curette, the following treatment protocols were performed.

### 2.2 Group I—Standard DL‐PDT

1. All exposed skin areas were covered with chemical sunscreen.
2. After 15 min, a uniform layer of methyl aminolevulinate (MAL) cream (Metvix, Galderma; 1 g) was applied to the face without occlusion.

### 2.3 Group II—DL‐PDT With Microneedling

1. All exposed skin areas were covered with chemical sunscreen.
2. After 15 min, a uniform layer of MAL cream (Metvix, Galderma; 1 g) was applied to the face without occlusion.
3. A motorized pen device with a tip containing 17 grouped 0.5‐mm needles (Dermapen Beauty, Korea) was applied in a single pass without inducing bleeding.

The procedure was intended to produce superficial microchannels through disruption of the stratum corneum and superficial epidermis. The exact number of needle impacts per unit area was not recorded, and therefore channel density per cm^2^ could not be determined.

### 2.4 Group III—DL‐PDT With Fractional CO_2_ Laser

1. All exposed skin areas were covered with chemical sunscreen.
2. After 15 min, excess sunscreen was removed, and ablative fractional CO_2_ laser treatment (AFXL) was performed using a roller‐type handpiece composed of one row of seven fractional pins (7 × 1) (Pixel, Alma Lasers). The laser parameters were: single pass, 60 W, 15 mJ/pixel, 125 μm/pixel, 2‐mm spacing between ablation zones, and density < 1%.
3. A uniform layer of MAL cream (Metvix, Galderma; 1 g) was applied to the face without occlusion.

These parameters produced discrete microscopic ablation zones separated by untreated skin. The reported density and spacing characterize the distribution of the ablation zones; however, the histological depth of the ablation columns was not directly measured in this study.

### 2.5 Group IV—DL‐PDT With Microdermabrasion

1. Microdermabrasion with aluminum oxide crystals (Pan Eletronic) was performed after superficial curettage. Three passes were applied in different directions (vertical, horizontal, and oblique) without inducing bleeding.
2. All exposed skin areas were covered with chemical sunscreen.
3. After 15 min, a uniform layer of MAL cream (Metvix, Galderma; 1 g) was applied to the face without occlusion.

Unlike microneedling and fractional CO_2_ laser, microdermabrasion does not create discrete vertical channels but produces mechanical removal of the stratum corneum and superficial epidermal disruption. The exact depth of tissue removal was not directly measured.

In all protocols, patients remained indoors for 30 min after the procedures and were subsequently exposed to daylight in an open environment for 2 h. Treatment sessions were not performed on rainy days or under conditions of heavy cloud cover that could substantially reduce sunlight exposure. After this period, the skin was cleansed with 0.9% saline solution and sunscreen was reapplied.

Daylight irradiance and cumulative light dose were not measured during treatment sessions because, according to the Latin American Consensus on DL‐PDT [7], routine irradiance measurements are not required when treatment is performed according to a standardized DL‐PDT protocol under appropriate weather conditions. Furthermore, a meteorological study demonstrated that evaluated locations in Central and South America, including Brazil, receive sufficient daylight irradiance to perform DL‐PDT throughout the year, provided that weather conditions allow comfortable 2‐h outdoor exposure [8].

The different timing of TED techniques in relation to MAL application was based on the specific mechanisms of action of each procedure. Microneedling was the only TED technique performed after MAL application, based on previous studies suggesting that blood and fluid flow into newly created channels could impair photosensitizer penetration if performed before MAL application. Additionally, the mechanical action of the needles may facilitate MAL penetration [9, 10]. The sequence of sunscreen application followed by fractional CO_2_ laser treatment was based on previously published DL‐PDT protocols combining ablative fractional laser as a TED technique with MAL. In these studies, chemical sunscreen was applied before the laser procedure, followed by MAL application, ensuring adequate photoprotection throughout the procedure [11, 12].

### 2.6 Immunohistochemical Analysis

Skin biopsies were obtained using a 5‐mm punch from the preauricular region without clinical AK lesions before treatment and 3 months after the second treatment session. The left side was designated as the pretreatment biopsy site, and the contralateral right side as the posttreatment biopsy site. The preauricular region was selected as a standardized and chronically sun‐exposed area within the field of cancerization. The entire face, including the preauricular region, was treated according to the assigned protocol. The second biopsy was performed at the contralateral site to minimize potential histological alterations related to wound healing and scar formation.

Skin sections were immunostained using the following primary antibodies: anti‐TP53 (clone DO‐7, 1:100; Dako, Denmark), anti‐Ki67 (clone SP6, 1:200; EasyPath, Brazil), anti‐collagen I and III (rabbit polyclonal; StressMarq Biosciences, Canada), anti‐MMP‐1 (clone EP1247Y, 1:100; Abcam, Cambridge, UK), anti‐MMP‐3 (polyclonal, 1:100; Abcam, Cambridge, UK), and anti‐MMP‐9 (clone 5G3, 1:200; Abcam, Cambridge, UK). Diaminobenzidine tetrahydrochloride (DAB) was used as the chromogen with the avidin–biotin peroxidase (ABC) method. All immunohistochemical analyses were performed by an investigator blinded to treatment allocation.

The expression of Ki67 and p53 was evaluated in all patients by determining the percentage of positive nuclei in the epidermis through manual counting. Digital morphometric quantification was not used because the software could not distinguish the immunohistochemical chromogen from epidermal melanin, as both present a brown coloration. Ki67 expression was also categorized according to the vertical extent of epidermal involvement (one‐third, two‐thirds, or full‐thickness involvement), based on parameters used for the classification of actinic keratosis [13].

Due to resource limitations, morphometric analysis of dermal marker expression, including Type I and III collagens, MMP‐1, MMP‐3, MMP‐9, and TIMP‐1, was performed in three randomly selected patients from each group using Apple's Random application (Mireia Lluch Ortola, version 1). Images were obtained using an Axioplan Zeiss microscope with a plan‐apochromatic lens at ×200 magnification and photographed with a Canon G10 14.7‐megapixel digital camera. Images were transferred to AxioVision 4.2 software (Carl Zeiss Industrielle Messtechnik GmbH) for area measurements, and quantification was performed using Image‐Pro Plus 3.2 software (Media Cybernetics, Rockville, Maryland, USA). Results were expressed as μm^2^, and the percentage relative to the total analyzed area was subsequently calculated.

### 2.7 Statistical Analysis

Statistical analyses were performed using the Wilcoxon test for comparisons before and after treatment within each group. Comparisons among treatment groups were performed using the chi‐square (*χ* ^2^) test or Fisher's exact test for categorical variables and the Kruskal–Wallis test for continuous variables. Spearman's and Pearson's correlation coefficients were used to assess associations between selected variables. Nonparametric tests were primarily used due to the limited sample size and the absence of assumptions of normal distribution for some variables. Statistical significance was set at *p* < 0.05. Data analysis was performed using SPSS (Statistical Package for the Social Sciences), version 21 (Armonk, New York, USA).

## 3 Results

All 40 patients completed the study. Of these, 31 (77.5%) were female and 9 (22.5%) were male, with a mean age of 62.8 ± 8.46 years. Twenty‐eight patients (70%) had Fitzpatrick Skin Phototype III, eight (20%) had Phototype IV, and four (10%) had Phototype II. According to the Glogau classification, 28 patients (70%) had Grade III Photoaging, eight (20%) had Grade IV, and four (10%) had Grade II.

The four treatment groups were comparable at baseline, with no statistically significant differences in sex (*p* = 0.436), age (*p* = 0.424), skin phototype (*p* = 0.746), Glogau photoaging grade (*p* = 0.377), previous history of skin cancer (*p* = 0.283), regular sunscreen use (*p* = 0.436), or the baseline number and severity of AK lesions (Table 1). Therefore, the groups were considered comparable at baseline.

**TABLE 1:** Demographic characteristics, medical history, and dermatological findings at baseline among the four treatment groups.

Variables	Group I	Group II	Group III	Group IV	*p*
Sex, female	8 (80%)	6 (60%)	9 (90%)	8 (80%)	0.439
Age, mean (years)	63.4	65.5	60.5	61.8	0.424
Fitzpatrick Phototype III	7 (70%)	8 (80%)	6 (60%)	7 (70%)	0.746
Glogau Photoaging Grade III	9 (90%)	5 (50%)	6 (60%)	8 (80%)	0.377
Previous history of skin cancer	7 (70%)	9 (90%)	7 (70%)	5 (50%)	0.283
Regular sunscreen use	8 (80%)	6 (60%)	9 (90%)	8 (80%)	0.436
Mean number of AK lesions	23	19	13.8	13.7	0.097
Mean number of Grade 1 AK lesions	11.6	10.9	6.7	6.6	0.099
Mean number of Grade 2 AK lesions	10.9	7.4	6.8	6.4	0.308
Mean number of Grade 3 AK lesions	0.5	0.7	0.3	0.7	0.403

*Note:* Group I: Standard DL‐PDT; Group II: DL‐PDT with microneedling; Group III: DL‐PDT with CO_2_ laser; and Group IV: DL‐PDT with microdermabrasion.

Abbreviations: AK, actinic keratosis; DL‐PDT, daylight photodynamic therapy.

Clinical outcomes were assessed at 1, 3, and 6 months after the second treatment session and have been previously reported [6]. AK clearance was significantly higher at 1 and 3 months in the CO_2_ laser group. Improvements in skin rejuvenation parameters, including skin texture, pigmentation, and fine lines, were more evident in the CO_2_ laser and microdermabrasion groups; however, these differences were not statistically significant.

Among the 40 patients evaluated for Ki67 expression, two patients in Group II were excluded because of absent immunostaining. In the remaining 38 patients, the percentage of Ki67‐positive cells increased in 25 patients and decreased in 13 patients (illustrated in Figures 1 and S1) 3 months after the second treatment session (*p* = 0.05). No significant differences were observed within individual treatment groups (Table 2) or among treatment groups (*p* = 0.706).

**FIGURE 1:**
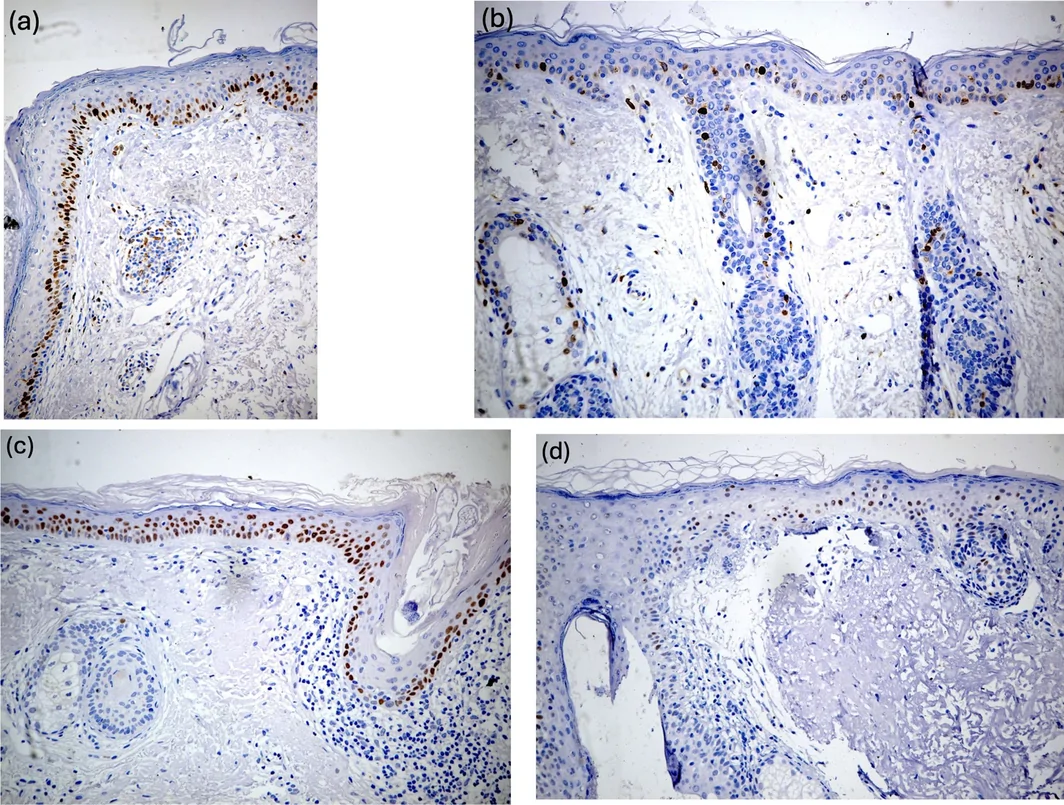
Reduction in the percentage and vertical extent of Ki67 expression in the epidermis (a) before treatment and (b) after 3 months. Reduction in the percentage of p53 expression in the epidermis (c) before treatment and (d) after 3 months. Group III patient (DL‐PDT with CO_2_ laser). Original magnification ×200.

**TABLE 2:** Median percentage of Ki67‐ and p53‐positive nuclei in the epidermis before and 3 months after the second treatment session, according to treatment group.

Group	Ki67‐positive nuclei, median % (range)			p53‐positive nuclei, median % (range)		
	Before	After	*p*	Before	After	*p*
I	10.0 (0.5–19.9)	10.8 (4.1–29.0)	0.241	0.5 (0–87.8)	4.7 (0–32.8)	0.767
II	5.9 (0.4–22.9)	11.5 (4.3–24.1)	0.484	0 (0–29.9)	0 (0–1.9)	0.080
III	8.8 (1.0–29.5)	11.4 (7.3–17.0)	0.721	6.9 (0–45.9)	4.1 (0–16.3)	0.214
IV	11.1 (0.5–21.9)	14.1 (4.7–26.3)	0.074	0.4 (0–9.6)	2.1 (0–25.9)	0.285

*Note:* Group I: Standard DL‐PDT; Group II: DL‐PDT with microneedling; Group III: DL‐PDT with CO_2_ laser; and Group IV: DL‐PDT with microdermabrasion.

Analysis of the vertical extent of Ki67 expression showed a reduction in cases with staining involving two‐thirds of the epidermis (47.4% to 36.8%) and an increase in cases with staining limited to the lower one‐third (52.6% to 63.2%), although these changes were not statistically significant (*p* = 0.503). This reduction in the vertical extent of Ki67 expression was observed mainly in the microneedling and CO_2_ laser groups (Table 3).

**TABLE 3:** Changes in the vertical extent of Ki67 immunostaining in the epidermis 3 months after the second treatment session, according to treatment group.

Group	Improvement^a^ or maintenance, *n* (%)	Worsening, *n* (%)	*p*
I	6 (60%)	4 (40%)	0.110
II	7 (87.5%)	1 (12.5%)	
III	10 (100%)	0	
IV	7 (70%)	3 (30%)	

*Note:* Group I: Standard DL‐PDT; Group II: DL‐PDT with microneedling; Group III: DL‐PDT with CO_2_ laser; and Group IV: DL‐PDT with microdermabrasion.

^a^ Reduction in the vertical extent of Ki67 immunostaining from two‐thirds to one‐third of the epidermis.

Analysis of p53 expression in all 40 patients showed a reduction (illustrated in Figures 1 and S2) in the percentage of positive nuclei in 17 cases, an increase in 16, and a maintenance of negative expression in seven cases; these changes were not statistically significant (*p* = 0.611). Likewise, no significant differences were observed within treatment groups (Table 2) or among treatment groups (*p* = 0.268).

A statistically significant positive correlation was observed between the baseline number of AK lesions and p53 expression in pretreatment biopsies (Pearson's correlation coefficient: *r* = 0.684, *p* = 0.001). A positive correlation was also observed using Spearman's rank correlation; however, it did not reach statistical significance (*r* = 0.255, *p* = 0.112).

For the dermal remodeling analysis, three patients from each treatment group were evaluated, totaling 12 patients. Overall, there was an increase in the median percentage of Type III collagen (illustrated in Figure 2), MMP‐3, and MMP‐9 (illustrated in Figure 3), together with a decrease in MMP‐1 and TIMP‐1 expression. The reduction in TIMP‐1 was the only statistically significant change (*p* = 0.041). No significant change was observed in Type I collagen (Table 4).

**FIGURE 2:**
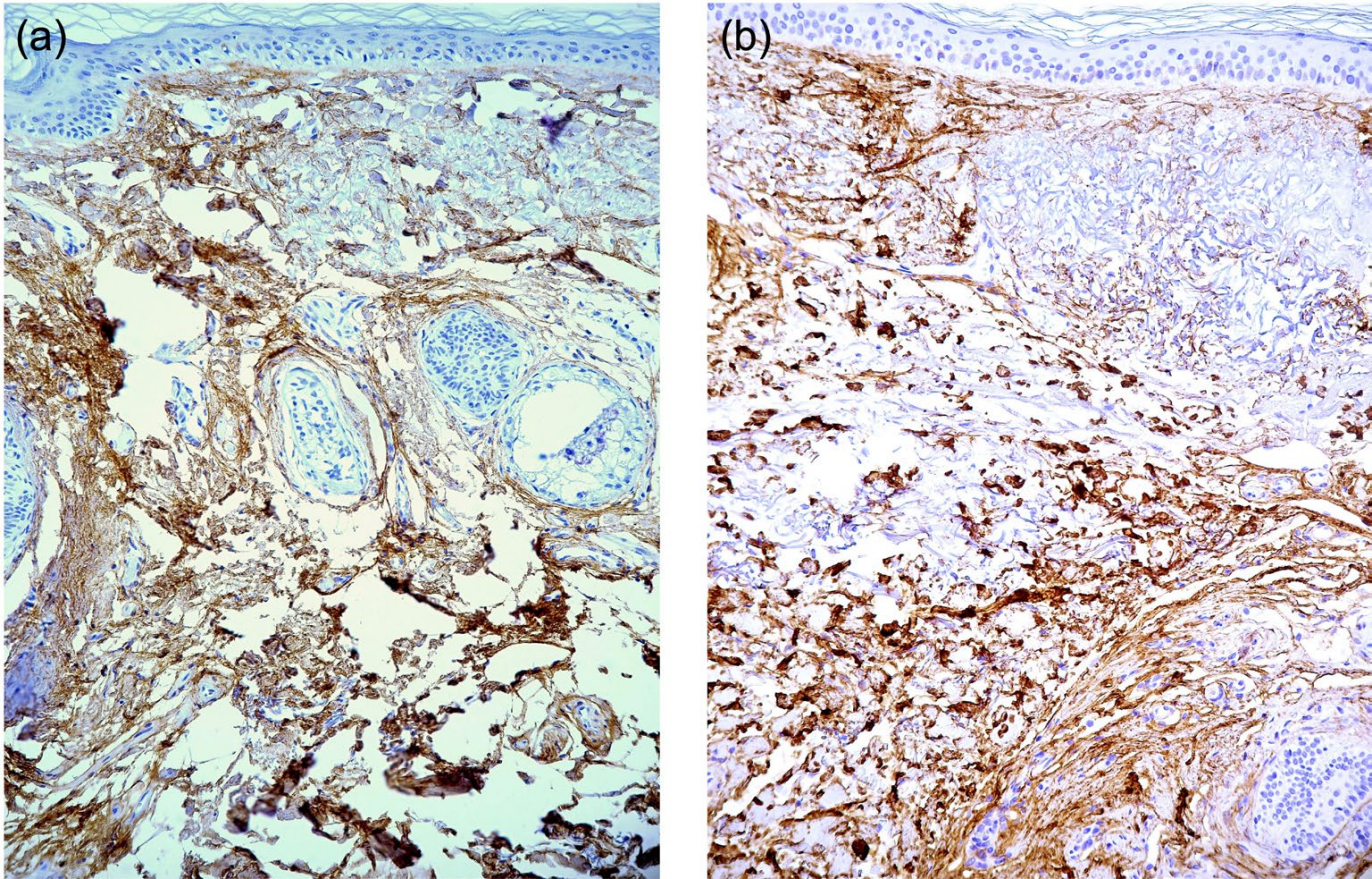
Increase in Type III collagen in a patient from Group II (DL‐PDT with microneedling) (a) before treatment (b) after 3 months. Original magnification ×200.

**FIGURE 3:**
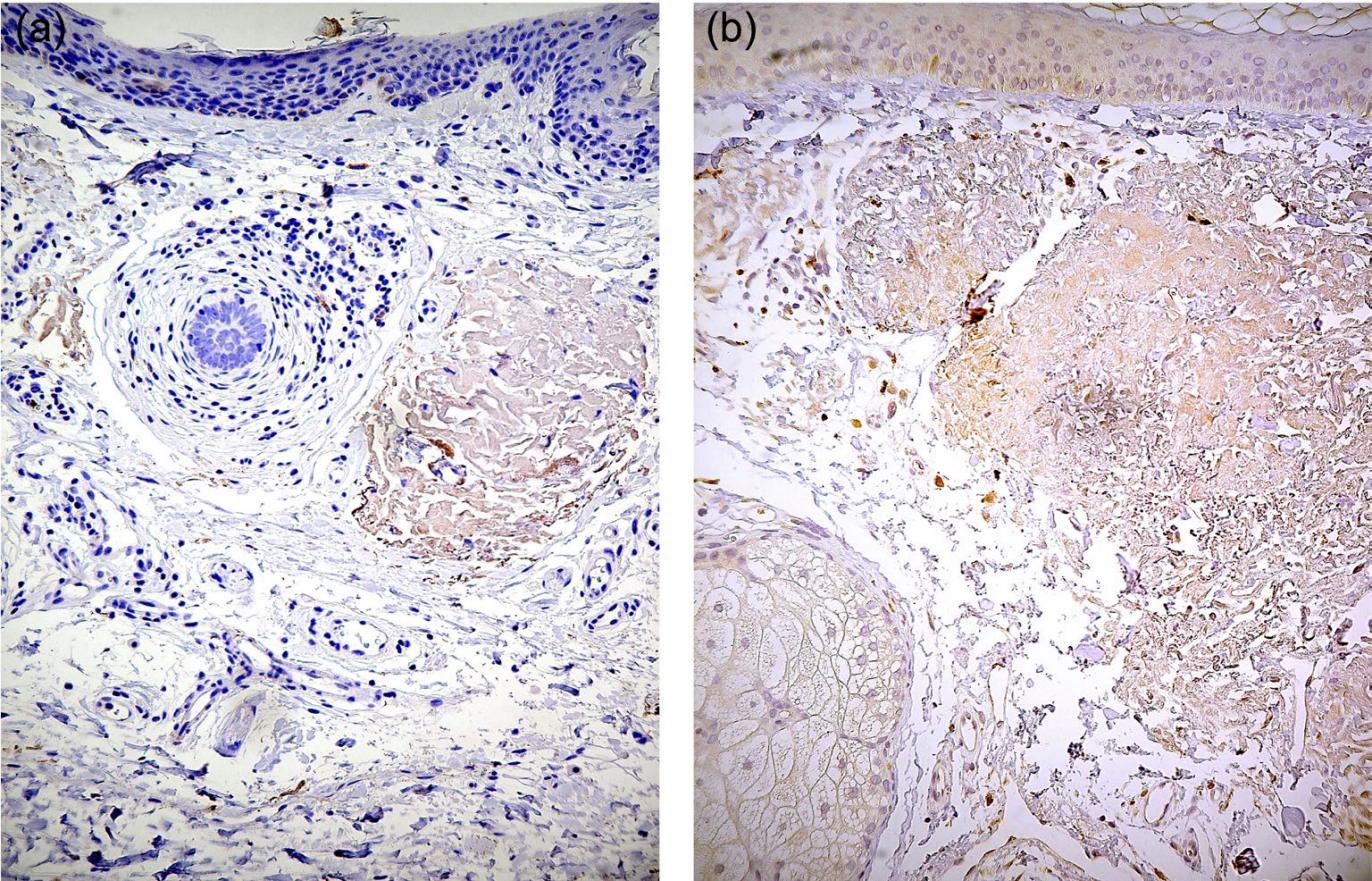
Increase in MMP‐9 in a patient from Group III (DL‐PDT with CO_2_ laser). (a) Before treatment and (b) after 3 months. Original magnification ×200.

**TABLE 4:** Median dermal marker expression before and 3 months after the second treatment session.

Marker	Before median % (range)	After median % (range)	*p*
Type I collagen	18.7 (4.4–54.3)	18.6 (9.0–29.8)	0.814
Type III collagen	14.0 (7.7–52.3)	25.9 (9.7–56.0)	0.239
MMP‐1	30.5 (10.4–54.3)	23.7 (9.4–41.9)	0.182
MMP‐3	14.8 (2.6–40.0)	16.4 (5.9–28.1)	0.754
MMP‐9	7.3 (0.3–20.4)	9.5 (1.2–40.9)	0.158
TIMP‐1	37.1 (26.2–53.6)	24.7 (8.4–62.0)	0.041

Abbreviations: MMP, metalloproteinase; TIMP‐1, tissue inhibitor of metalloproteinases‐1.

## 4 Discussion

This is the first study to evaluate epidermal carcinogenesis and dermal remodeling markers by immunohistochemistry after DL‐PDT alone or combined with different TED techniques in patients with skin field cancerization. We observed a statistically significant positive correlation between the baseline number of AK lesions and p53 expression in pretreatment biopsies, supporting the association between the extent of clinical field damage and p53 expression in the field cancerization.

Previous experimental studies demonstrated that different TED techniques can result in different degrees of protoporphyrin IX (PpIX) accumulation, with ablative fractional CO_2_ laser producing greater fluorescence than microneedling and microdermabrasion [14]. Importantly, Bay et al. found that increasing laser density from 2% to 6% or increasing the number of pretreatment passes from one to three did not further increase PpIX fluorescence or photobleaching, suggesting that greater epidermal disruption does not necessarily result in proportional increases in photosensitizer accumulation or photodynamic response [14]. Nissen et al. similarly demonstrated that increased PpIX accumulation did not improve the clinical efficacy of PDT for AKs [15]. These findings suggest that, once sufficient photosensitizer is available to generate an effective photodynamic response, further increases in PpIX accumulation may not necessarily translate into greater therapeutic benefit.

The purpose of comparing these techniques in the present study was to evaluate clinically applicable TED approaches with different mechanisms of epidermal barrier disruption, rather than to assume equivalent MAL penetration. MAL penetration and PpIX accumulation were not directly measured in the present study; therefore, differences in photosensitizer delivery among the TED techniques cannot be excluded. The clinical and histological outcomes of these treatment protocols have been previously reported [6]. The present study extends these findings by evaluating whether the different TED‐assisted DL‐PDT protocols were associated with differential effects on epidermal carcinogenesis and dermal remodeling biomarkers.

Lower percentages of epidermal marker expression (Ki67 and p53) were observed in our study compared with previous studies evaluating field cancerization [16, 17]. However, in those studies, biopsies were obtained from the forearms adjacent to AK lesions, whereas our biopsies were performed in clinically unaffected preauricular facial skin, regardless of the location of AK lesions. Differences in biopsy sites and immunohistochemical assessment methods may therefore explain these findings. Similarly, a study evaluating preauricular biopsies, as performed in the present study, reported lower mean percentages of p53 expression (5.64 before and 5.44 after MAL‐PDT) [18].

We expected a reduction in the expression of the carcinogenesis‐related markers Ki67 and p53 after treatment. Importantly, although biopsies were obtained from clinically normal‐appearing skin, histological examination of the pretreatment specimens in our previous analysis of the same study population and biopsy specimens demonstrated epidermal atypia in 97.5% of cases [6]. This finding supports the presence of subclinical carcinogenesis‐related alterations in the clinically unaffected‐appearing preauricular skin and reinforces the rationale for evaluating Ki67 and p53 as markers of biological changes within the cancerized field. Nevertheless, differences in biopsy location (AK lesions versus clinically unaffected‐appearing field cancerization areas) and the lack of standardization among immunohistochemical assessment methods may have contributed to differences in the magnitude of Ki67 and p53 expression reported across studies. Bagazgoitia et al. demonstrated reductions in Ki67 and p53 expression 6 weeks after a single session of MAL‐PDT; however, biopsies were obtained directly from AK lesions and evaluated using a semiquantitative tissue scoring system [19]. In contrast, Szeimies et al., who evaluated biopsies from field cancerization areas, as in the present study, observed a nonsignificant reduction in p53 expression 3 months after three sessions of MAL‐PDT, while Ki67 was not evaluated [18]. Abdalla et al. assessed both AK lesions and clinically unaffected field cancerization areas 2 months after a single DL‐PDT session and demonstrated a reduction in Ki67 expression that reached statistical significance only in biopsies from field cancerization areas, whereas no reduction in p53 positivity was observed [20]. Taken together, these findings suggest that the effects of PDT on epidermal carcinogenesis‐related markers may vary according to the tissue sampled, treatment protocol, timing of biopsy, and method used for immunohistochemical quantification.

In the present study, although no significant reduction in the percentage of Ki67‐positive cells was observed, we identified a shift in the vertical distribution of Ki67 expression, with a reduction in cases showing involvement of two‐thirds of the epidermis and an increase in cases restricted to the lower one‐third. This change was observed mainly in the microneedling and CO_2_ laser groups. Since Ki67 expression is normally restricted to basal keratinocytes in healthy epidermis, its reduced extension toward suprabasal layers may indicate a favorable alteration in epidermal proliferative activity [21]. However, this finding should be interpreted cautiously due to the exploratory nature of the analysis and the absence of statistical significance.

Regarding p53 expression, no statistically significant differences were observed after treatment within or among the treatment groups. Although p53 immunoreactivity is frequently associated with TP53 mutations, positive staining may occur in the absence of mutations, and mutations may also occur without detectable immunohistochemical expression [22]. Therefore, p53 immunohistochemical expression should be interpreted as an indirect marker of cellular alterations rather than a direct assessment of TP53 mutation status.

Regarding dermal remodeling markers, an increase in Type III collagen expression was observed, whereas Type I collagen remained unchanged. This finding may be related to the timing of biopsy collection, performed 3 months after treatment, since Type III collagen is synthesized early during dermal remodeling and is subsequently replaced by Type I collagen during the maturation process [23]. We also observed increased expression of MMP‐3 and MMP‐9, together with reduced MMP‐1 expression. These findings are consistent with the expected remodeling process, in which MMPs participate in the degradation of damaged extracellular matrix components and facilitate subsequent collagen remodeling [3, 24].

Issa et al. found no differences in TIMP‐1 expression 3 months after conventional PDT [3]. In the present study, a reduction in TIMP‐1 expression and an increase in MMP‐9 expression were observed after DL‐PDT. TIMP‐1 inhibits several MMPs, particularly MMP‐9 [25]. Therefore, reduced TIMP‐1 expression may contribute to increased MMP‐9 activity, potentially favoring degradation of altered collagen fibers and subsequent extracellular matrix remodeling. However, these findings should be interpreted with caution because dermal markers were evaluated in a limited number of patients.

## 5 Limitations

This study has some limitations. First, it was an exploratory study based on a convenience sample of 40 patients randomized into four treatment groups (*n* = 10 per group), which limited the statistical power to detect differences among groups. Although all patients were included in the analysis of epidermal biomarkers, dermal remodeling markers were evaluated in only three randomly selected patients per group due to resource limitations, which further reduced the statistical power and may limit the generalizability of these findings. Second, multiple biomarkers were evaluated, and although no significant overall differences among treatment groups were identified that would warrant post hoc multiple comparison analyses, the exploratory nature of these analyses requires cautious interpretation of the findings. Therefore, the results should be considered exploratory and require confirmation in larger, adequately powered randomized studies.

## 6 Conclusions

DL‐PDT, alone or with TED techniques, was associated with changes in immunohistochemical markers of epidermal carcinogenesis and dermal remodeling. However, no significant differences among treatment groups were observed for most outcomes. A nonsignificant reduction in the vertical extent of Ki67 expression, particularly in the microneedling and CO_2_ laser groups, and favorable changes in dermal remodeling markers were observed in the overall analysis. These findings are exploratory, particularly considering the limited sample size for dermal analyses, and require confirmation in larger, adequately powered randomized studies.

## Funding

This study was conducted with support from the Coordenação de Aperfeiçoamento de Pessoal de Nível Superior—Brasil (CAPES)—Finance Code 001. Galderma Industry donated Metvix and sponsored some of the immunohistochemical markers; however, the company had no role in the study design, data collection, analysis, manuscript preparation, or publication decisions.

## Ethics Statement

The study was approved by the Research Ethics Committee of Antônio Pedro University Hospital under approval number 1.901.583.

## Consent

Written informed consent for publication was obtained from all participants.

## Conflicts of Interest

The authors declare no conflicts of interest.

## Supporting information


**Figure S1:** Representative immunohistochemical images from standard, microneedling, fractional CO_2_ laser, and microdermabrasion groups. Reduction in the percentage and vertical extent of Ki67 expression in the epidermis (a, c, e, g) before treatment and (b, d, f, h) after 3 months. Original magnification ×200.

**Figure S2:** Representative immunohistochemical images from standard, microneedling, fractional CO_2_ laser, and microdermabrasion groups. Reduction in the percentage of p53 expression in the epidermis (a, c, e, g) before treatment and (b, d, f, h) after 3 months. Original magnification ×200.

## Data Availability

The data that support the findings of this study are available from the corresponding author upon reasonable request.
